# 10-hydroxy-2E-decenoic acid (10HDA) does not promote caste differentiation in *Melipona scutellaris* stingless bees

**DOI:** 10.1038/s41598-021-89212-5

**Published:** 2021-05-10

**Authors:** Luiza Diniz Ferreira Borges, Letícia Leandro Batista, Serena Mares Malta, Tamiris Sabrina Rodrigues, Jéssica Regina da Costa Silva, Gabriela Venturini, Alexandre da Costa Pereira, Pedro Henrique Gonçalves Guedes, Carlos Ueira-Vieira, Ana Maria Bonetti

**Affiliations:** 1 Institute of Biotechnology, Federal University of Uberlândia, Acre Street, 2E building, Uberlândia, MG 38405-319 Brazil; 2 Department of Genetics, Harvard Medical School, Boston, MA USA

**Keywords:** Epigenetics, Entomology, Metabolomics

## Abstract

In bees from genus *Melipona*, differential feeding is not enough to fully explain female polyphenism. In these bees, there is a hypothesis that in addition to the environmental component (food), a genetic component is also involved in caste differentiation. This mechanism has not yet been fully elucidated and may involve epigenetic and metabolic regulation. Here, we verified that the genes encoding histone deacetylases HDAC1 and HDAC4 and histone acetyltransferase KAT2A were expressed at all stages of *Melipona scutellaris*, with fluctuations between developmental stages and castes. In larvae, the HDAC genes showed the same profile of Juvenile Hormone titers—previous reported—whereas the HAT gene exhibited the opposite profile. We also investigated the larvae and larval food metabolomes, but we did not identify the putative queen-fate inducing compounds, geraniol and 10-hydroxy-2E-decenoic acid (10HDA). Finally, we demonstrated that the histone deacetylase inhibitor 10HDA—the major lipid component of royal jelly and hence a putative regulator of honeybee caste differentiation—was unable to promote differentiation in queens in *Melipona scutellaris*. Our results suggest that epigenetic and hormonal regulations may act synergistically to drive caste differentiation in *Melipona* and that 10HDA is not a caste-differentiation factor in *Melipona scutellaris*.

## Introduction

In the majority of social insects, female castes share the same genome; however, highly reproductive long-lived queens and facultatively sterile short-lived workers contrast in morphological, physiological, and behavioural traits. This fascinating biological phenomenon, in which one genotype produces more than one phenotype, is referred to as polyphenism^1–4^. It occurs in response to different stimuli: environmental (chemical, nutritional, physical, etc.); physiological; or development-related.

The mechanisms that promote caste differentiation in stingless bees have not yet been completely elucidated. Different from most Hymenoptera, in which this process is nutritionally driven, in stingless bees from the genus *Melipona*, an interaction between genetic and nutritional factors is proposed^5^. In *Melipona*, the supply of food in the brood cells is massive; queens, workers, and males develop in undifferentiated brood cells, with the same size and the same quantity of food^5–7^. In addition, during larval development it is impossible to distinguish caste or sex, which makes studies on caste differentiation limited to some protocols.

The model of genetic-feeding control for caste differentiation in *Melipona* was proposed by Kerr, in 1950, to explain the fact that under optimal colony conditions, up to 25% of total females develop in queens. According to this mechanism, the divergences between queens and workers would be determined by the presence of two genes, with two alleles each. In conditions that the larva ingested enough food, the double heterozygosis would lead to higher titers of juvenile hormone (JH), and, therefore, result in the differentiation of the larva into queen. Homozygosis for one or both genes would lead to the development of workers, regardless of the amount of food received^5,6,8^.

It is generally accepted that JH, an acyclic sesquiterpenoid hormone, is the main hormone that regulates caste differentiation and age polyethism in bees^9–11^. Topical application of JH in *Melipona’s* third instar larvae promotes differentiation into queens^9,12,13^. Additionally, a previous study suggested that the addition of geraniol, a compound of labial secretion of nurse bees, to larval food is another key factor for caste differentiation in *Melipona*. According to these authors, this compound can promote differentiation in queens, in larvae with genetic predisposition, reaching the percentage of 25% of queens predicted by the KERR hypothesis^14^. Our group has found evidence of olfactory function in *Melipona scutellaris* larvae^15^, indicating that environmental stimuli, such as geraniol^14^ or other larval food components, could be perceived by this olfactory system, triggering cellular responses involved in caste differentiation in these bees.

Currently, it is largely recognized that epigenetic mechanisms are associated with caste polyphenisms regulation^16–29^. Honeybees and bumblebees present different sets of microRNAs^30–35^ as well as differences in methylation^16,29,36–41^ according to the caste. Histone post-translational modifications were described in *Apis mellifera*^42,43^. In this species, royal jelly is composed of up to 6% of 10-hydroxy-2E-decenoic acid (10HDA), a broad-spectrum histone deacetylase inhibitor (HDACi). 10HDA is potentially important in the regulation of genes triggering queen differentiation^18,44^. The presence of epigenome-modifying compounds—such as 10HDA—and microRNAs in larval food may indicate a role of nutrition in driving epigenetic patterns related to development and caste in these bees^20,44–46^.

Concerning stingless bees, works on their epigenetics remain limited. Recently, a complete set of genes involved in DNA methylation and in histone post-translational modifications in the genome of *Frieseomellita varia* was reported^47^. Moreover, in our previous studies, we demonstrated a functional methylation/demethylation system as well as post-translational modifications of histones in the stingless bee *Melipona scutellaris*. We found that both the levels of phosphorylation at threonine 3 of histone H3 (H3T3-P) and the levels of monomethylation at lysine 4 of histone H3 (H3K4-Me) are higher in newly emerged queens than in workers at the same age^48^. Analysing *corpora allata* glands, which are responsible for JH production, we have shown that the territorial dispersion of heterochromatin could be an important epigenetic mechanism associated with the phenotypic plasticity in castes of this species^49^.

Histone acetylation is controlled by the balance of activities of two enzyme families: histone acetyltransferases (HATs)—which acetylate lysine residues of histones, promoting gene expression—and histone deacetylases (HDACs)—which remove these Ɛ-acetyl-lysine residues, promoting condensation of chromatin and reduction in gene expression^50–52^. In the current study, we investigate the importance of histone acetylation and nutritional factors to caste differentiation in *Melipona*. First, we verified that the mRNA levels of three genes related to the acetylation machinery during *Melipona scutellaris* development (*hdac1*, *hdac4,* and *kat2a*) fluctuate according to developmental stages and castes and are related to JH titers. In addition, when examining the larval food composition and the metabolic profile of *Melipona scutellaris* larvae, we did not identify the putative queen-fate inducing compounds, geraniol and 10HDA. Finally, topical application of 10HDA in *Melipona scutellaris* larvae was unable to promote differentiation in queens. Taken together, our data indicate that epigenetic and hormonal regulations may act synergistically to drive caste differentiation in *Melipona* and that 10HDA is not a caste-differentiation factor in *Melipona scutellaris*.

## Results

### Expression of histone acetyltransferase and histone deacetylase genes are temporally related to JH titers in larvae

**Figure 1:**
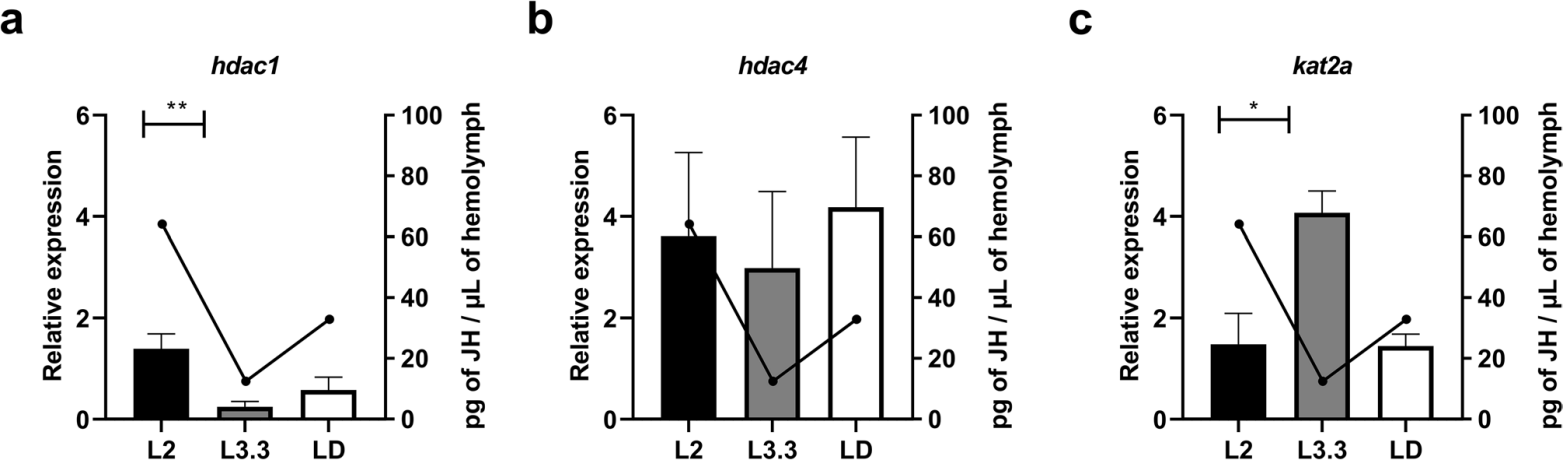
Comparison between expression of HDACs and HAT genes and JH titers in *Melipona scutellaris* larvae. The graphs show the overlapping of relative expression of *hdac1* (**a**), *hdac4* (**b**), and *kat2a* (**c**) quantified by RT-qPCR and JH titers quantified by radioimmunoassay. L2 = larva of the second stage; L3.3 = larva of the third stage in the third instar; LD = defecating larva. The graphs show mean ± SEM (n ≥ 5). Statistical analysis: Kruskal–Wallis test with a post-hoc Dunn’s multiple comparisons test, P < 0.05. JH data are from the work of Cardoso et al.^53^.

Quantitative real-time PCR was used to analyse the expression of two HDACs encoding genes (*hdac1* and *hdac4*) and one HAT encoding gene (*kat2a*) in the three main critical developmental stages of *M. scutellaris* larvae (L2, L3.3, and LD) (Fig. 1). Among the seven larval stages presented by *M. scutellaris*, these stages were analysed because: L2 larvae have the highest JH titers in larvae; L3.3 larvae represent the temporal window in which *M. scutellaris* larvae are sensitive to juvenile hormonal application; and LD represent the end of larval development. The expression of both HDAC genes had a decrease in transcripts levels from L2 to L3.3 stages, which was significative only for *hdac1* (Dunn’s multiple comparisons test, P = 0.0051) but not for *hdac4*. The expression of both genes was followed by an increase in LD stage. Interestingly, we noticed that this expression pattern (high in L2 larvae, lower in L3.3 larvae, and slightly higher in LD larvae) presents the same modulation of JH described by Cardoso et al.^53^. The *kat2a* transcripts presented an inverse expression pattern compared to HDAC genes and JH levels^53^, which significantly rises from L2 to L3.3 (Dunn’s multiple comparisons test, P = 0.042) and decreases in the LD stage. The JH data^53^ were used to build the graphs presented in Fig. 1—overlapping the JH titers and the expression levels of HDAC and HAT genes—in order to clarify the relationship between hormonal curves and the genes studied in the present work.

### Histone deacetylases genes show caste-specific expression patterns

**Figure 2:**
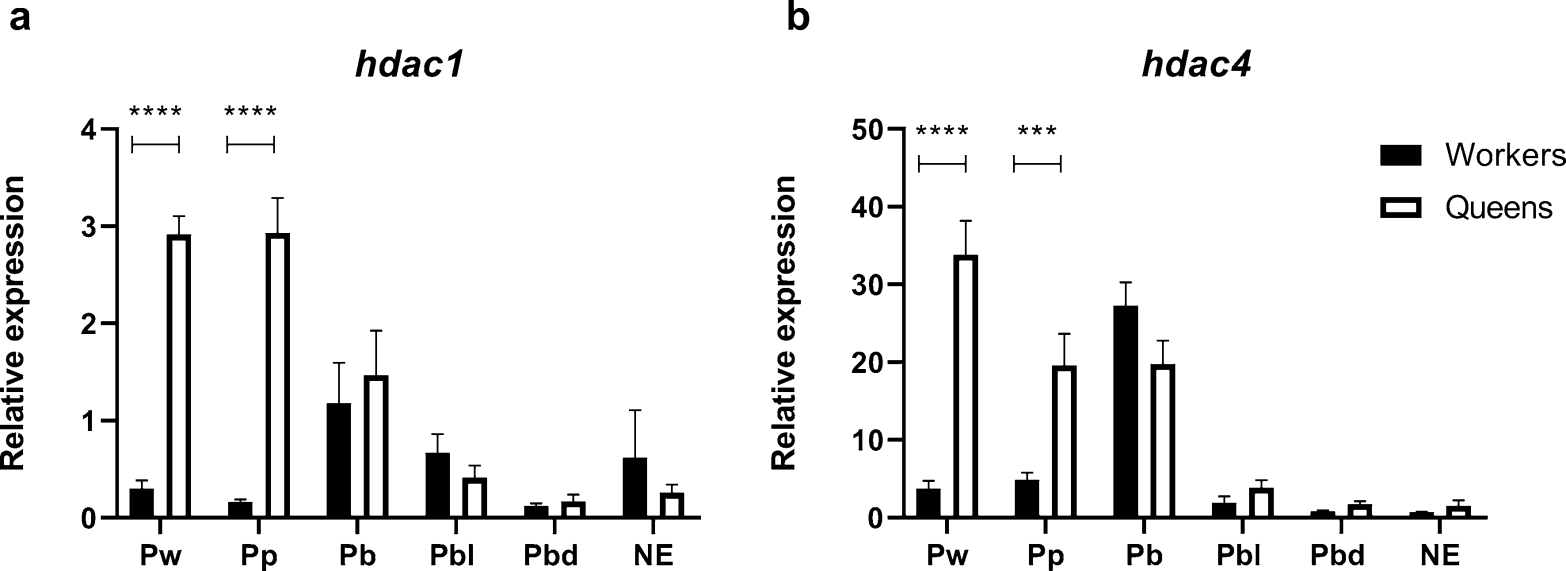
Expression of HDACs genes in *Melipona scutellaris* workers and queens. Relative expression of *hdac1* (**a**) and *hdac4* (**b**) quantified by RT-qPCR. Pw = pupa with white body and eyes; Pp = pupa with white body and pink eyes; Pb = pupa with white body and brown eyes; Pbl = pupa with light pigmented body and brown eyes; Pbd = pupa with dark pigmented body and brown eyes; NE = newly emerged adult. The graphs show mean ± SEM (n ≥ 3). Statistical analysis: 2Way-ANOVA with a post-hoc Sidak's multiple comparisons test, P < 0.05.

The mRNA levels of *hdac1*, *hdac4,* and *kat2a* genes was also evaluated during *M. scutellaris* pupal development, in which external morphological differences associated with caste and sex are distinguishable, and in newly emerged adults in both female castes (Fig. 2). For *kat2a* transcript levels, no significative difference between workers and queens was found (data not shown). Differentially, both HDAC genes had higher expression levels in queens than in workers at the first two pupae stages—Pw (Sidak's multiple comparisons test, P < 0.0001, for *hdac1* and *hdac4*) and Pp (Sidak's multiple comparisons test, P < 0.0001, for *hdac1* and P = 0.0004, for *hdac4*).

### Metabolomic profiles of larvae bees

The fact that the epigenome is sensitive to the metabolic state^54–57^ led us to investigate whether there are metabolic changes along the larval development of *M. scutellaris* related to epigenetic mechanisms and hormonal signalling, which may be associated with caste differentiation.

**Figure 3:**
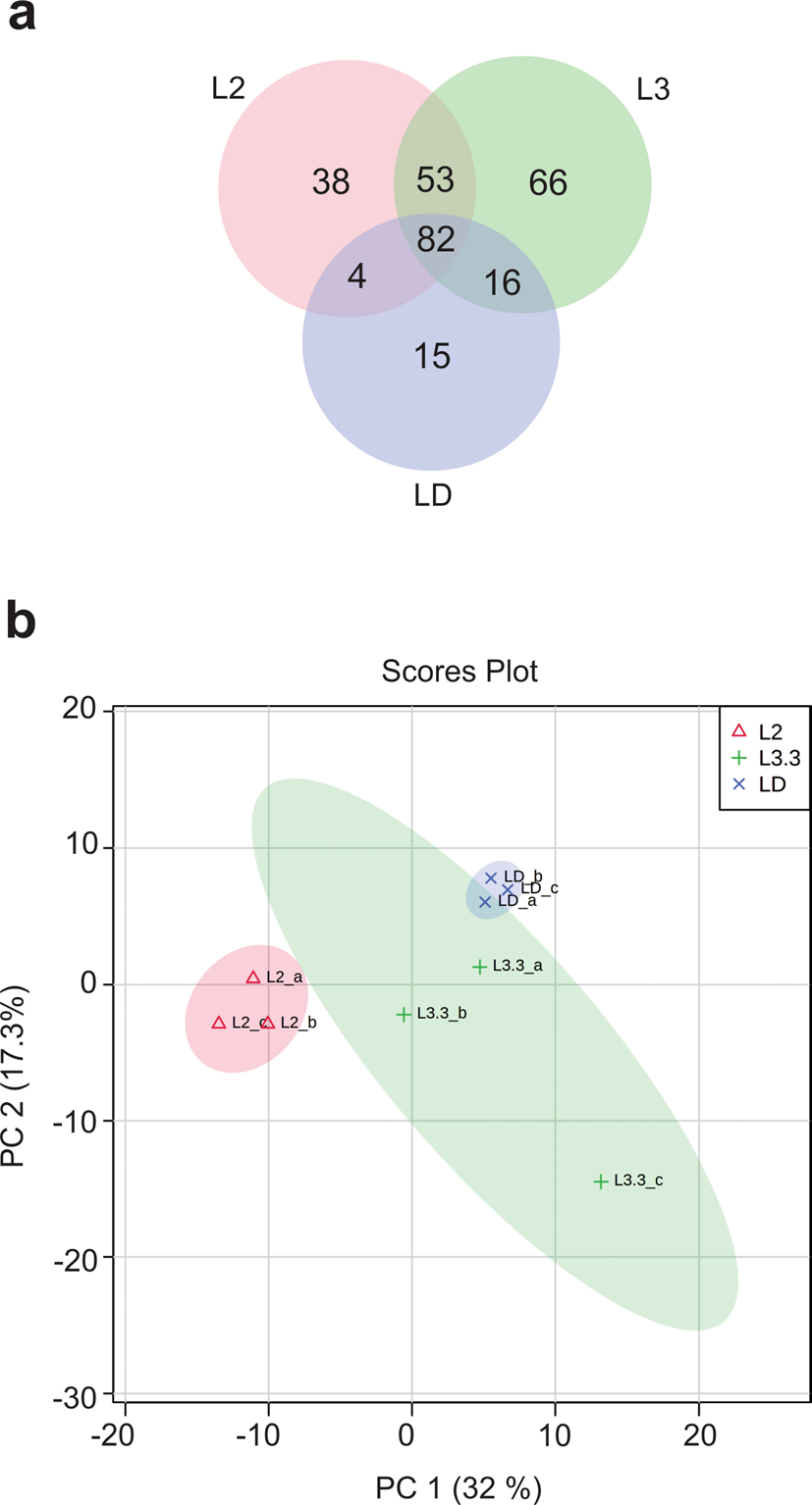
Metabolomic profiles of *Melipona scutellaris* larvae. Venn diagram of shared metabolites between larval stages (**a**) and PCA clustering of samples (**b**). L2 = larva of the second stage; L3.3 = larva of the third stage in the third instar; LD = defecating larva.

For the first time, to our knowledge, the metabolites present during the larval development of one stingless bee were tentatively identified and quantified by CG-MS*.* We tentatively identified 274 metabolites (Supplementary Table S1) with concentrations that vary among larval stages (L2, L3.3, and LD) and same stage samples. Significance Analysis of Microarray (SAM) identified 45 metabolites significantly different between the larval stages (Supplementary Table S2 and Supplementary Figure S1). Among the 274 metabolites, 38 were found exclusively in L2, 66 in L3.3, and 15 in LD (Fig. 3a).

**Figure 4:**
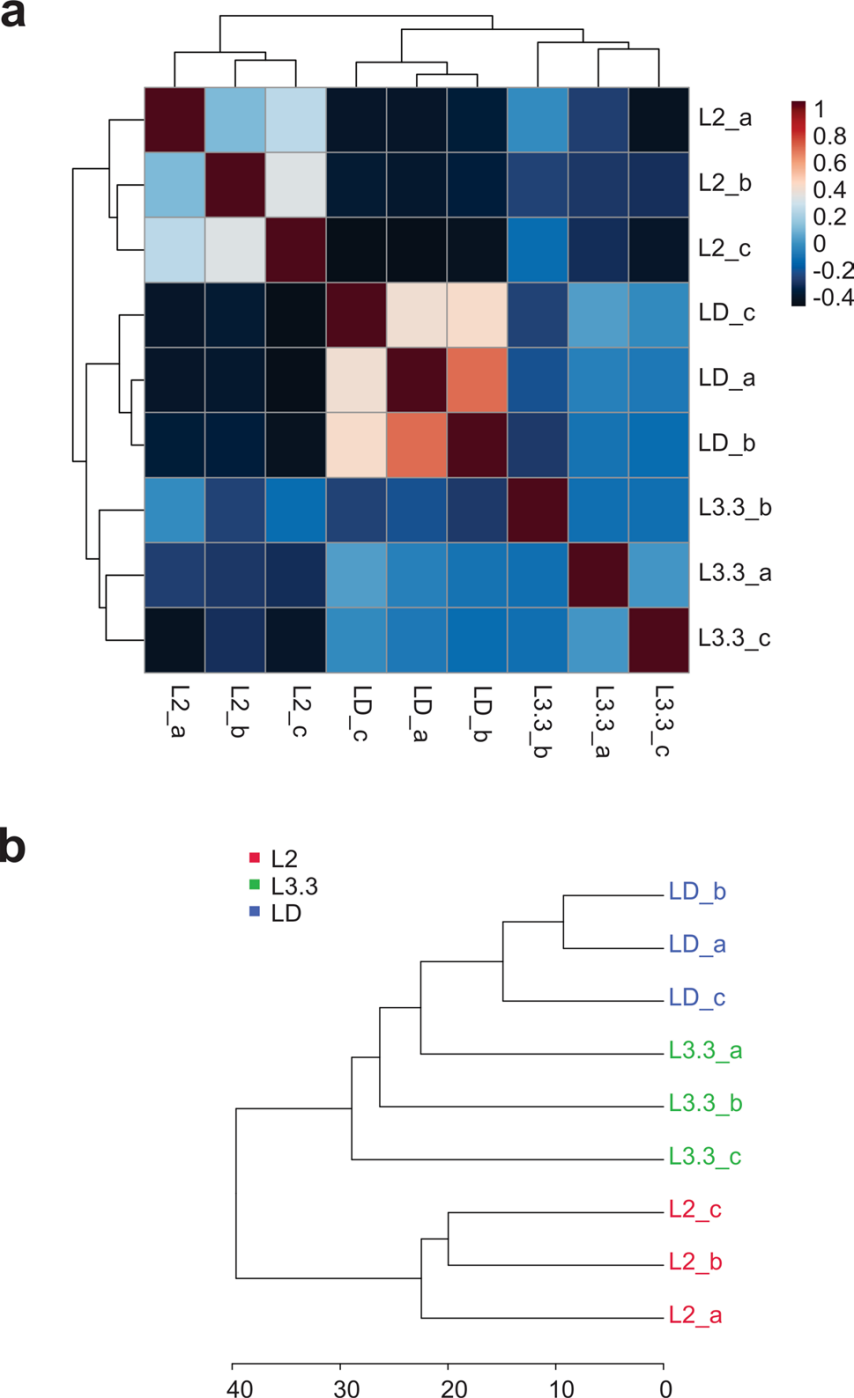
Correlation of metabolomic profiles of each sample of *Melipona scutellaris* larvae. Correlation heatmap of samples (**a**) and Hierarchical Clustering Dendrogram of samples (**b**). L2 = larva of the second stage; L3.3 = larva of the third stage in the third instar; LD = defecating larva.

Principal Component Analysis (PCA) was able to discriminate the samples from the three analysed larval stages, L2, L3.3, and LD, with stage L3.3 presenting samples with more distinct metabolites profiles (Fig. 3b). Likewise, correlation analysis indicates a very low correlation between the L3.3 samples (Fig. 4a). Both the correlation analysis and the dendrogram show that individuals from L3.3 and LD stages presented a closer metabolic profile than between each of these stages and L2 larvae (Fig. 4a and 4b).

The enrichment analysis indicates that for all analysed larval stages, there was significant enrichment for metabolites related to ammonia recycling and the urea cycle (P < 0.05). For L2 and L3.3 stages, there is also enrichment for galactose metabolism. Furthermore, L3.3 samples show enrichment for amino acids metabolisms, and LD for amino acids and purines metabolisms and for Warburg effect (Supplementary Table S3). The significantly enriched pathways, identified using *Drosophila melanogaster* KEGG database, are indicated in Supplementary Table S4.

### Metabolites of larval food

We tentatively identified 369 metabolites in *M. scutellaris* larval food (Supplementary Table S5), using GC–MS analysis. Neither geraniol nor 10HDA were identified in the larval food of *Melipona* bees. Only two metabolites (glycerol and 2,3-butanediol) were found in all samples (Supplementary Figure S2). There was significative enrichment for metabolites related to lactose degradation and galactose metabolism (P < 0.05) (Supplementary Table S6).

### 10HDA-treatment induces mortality and does not promote caste differentiation

**Figure 5:**
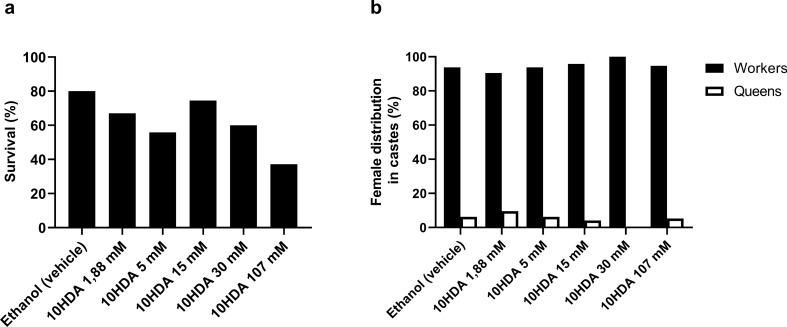
Effects of treatment of *Melipona scutellaris* on the third larval instar with 10HDA. Survival rate (**a**) and Distribution of females in castes, queen and worker (**b**).

*M. scutellaris* larvae in stages L3.3 and LPD (third larval instar) were topically treated with 10HDA, a broad-spectrum inhibitor of class I and II HDACs, at 1.88 mM; 5 mM; 15 mM; 30 mM and 107 mM. None of the five concentrations tested was able to induce differentiation of the larvae in queens and the highest concentration (107 mM) caused high mortality (62.77%) (Fig. 5).

## Discussion

Genes are not always turned on or off in one of the castes, they can be differentially expressed, at low, moderate or high levels. This indicates that a subtle regulation of metabolic steps and differential expression of genes affect reproductive capacity and the different behaviour patterns presented by queens and workers^58^. Here, we verified the transcripts level of *hdac1*, *hdac4,* and *kat2a* genes in larvae, pupae, and newly emerged adults of *M. scutellaris*. The fluctuations in the expression levels among developmental stages and castes reinforce this hypothesis of the subtle regulation of the caste differentiation process. Interestingly, in our metabolomics analysis, we were not able to identify the putative queen-fate inducing compounds described for other bee species—geraniol for *Melipona beecheii*^14^ and 10HDA for *Apis mellifera*^18,44^.

Expression of HDAC encoding genes have not been studied during development in bees, however they were extensively investigated in beetles^59–61^. We detected higher levels of *hdac1* transcripts in the beginning of larval development, followed by a decrease in the intermediate stage, and by a subsequent increase in the last larval stage. Similar profiles of mRNA relative expression levels of *hdac1* had been previously described in *Tribolium castaneum*, in which HDAC1 expression is essential to larval survival^59,61^. In this beetle species, this gene presents higher transcripts levels in eggs and 1-day-old larvae and lower levels during larval development. The expression starts to increase in the late-larvae stages and reach a dramatic increase in 1-day-old pupae^59^, as we also observed for queen but not for worker pupae in *M. scutellaris*. In addition, recently, it was suggested a role for HDAC1 in embryogenesis and in eclosion in pea aphids^62^. All together, these evidences indicate that HDAC1 may be potentially involved in insects development.

Extensive research demonstrates that many biological factors—such as pheromones, hormones and differentially expressed genes—are necessary to reach the regulatory complexity of caste differentiation^63^. Accordingly, we have shown that the expression of *hdac1* presents an opposite profile from *kat2a*, and these patterns are temporally related to JH titers in larvae^53^. This suggests that epigenetic and hormonal mechanisms may act synergistically underlying gene regulation in *M. scutellaris* larvae.

The higher expression of *hdac1* and *hdac4* transcripts in the early pupal stages of *M. scutellaris* queens may represent a layer of regulation of caste-specific transcriptional programs. If their expression patterns are coherently associated with their protein products, queens may suffer a remotion of Ɛ-acetyl-lysine residues from part of their histones, promoting local chromatin condensation and punctual and refined control of gene silencing. In agreement with our gene expression data, it was demonstrated in honeybees that aged-matched workers and queens exhibit distinct patterns of histone post-translational modifications^42,43^, including caste-specific regions of intronic H3K27ac marks identified in the worker caste^43^.

The differences in the expression of the HAT and HDACs genes supported our decision to investigate possible metabolic alterations along with the larval development of *M. scutellaris* related to epigenetic mechanisms and hormonal signalling. We were able to cluster samples in their respective larval stage according to their metabolic profiles. Samples from stage L3.3 presented more intergroup variability than samples from stages L2 and LD. This can be explained by the fact that it is impossible to identify sex or caste in *Melipona* larvae, being feasible to assume that L3.3 samples are from different sex or caste. In addition, L3.3 is the developmental stage in which JH acts, promoting caste differentiation^13^. Thus, it is possible that differences in JH titers, may lead to different metabolites sets, if these samples really are from different sex or caste.

All larval stages were mainly enriched for metabolites related to carbohydrates and amino acids metabolisms. This is not surprising as the larval food—the only nutrient source for these larvae—is a complex mixture of fermented pollen (protein), honey (carbohydrate) and hypopharyngeal glandular secretions^64,65^. In addition, the enrichment for amino acids and purine metabolisms in the end of the third larval instar can be associated with tissue remodelling process, which occurs in this stage to promote metamorphosis.

Despite some exclusive metabolites found in each stage, alone, these molecules could not be linked to epigenetic mechanisms or hormonal signalling. Furthermore, among the 274 metabolites tentatively identified in GC–MS, no metabolites directly linked to geraniol and 10HDA metabolisms were identified. Jarau et al.^14^ have shown that the addition of geraniol, a compound present in *M. beecheei* labial gland secretions, to larval food increases the number of larvae that develop in queens. Plus, 10HDA, the main fatty acid in royal jelly from *A. mellifera*, is a molecule with a potential role in caste differentiation in this species^18,44^. Our metabolomics data indicate, however, that geraniol and 10HDA may not act as queen-inducing factors in *M. scutellaris.*

To confirm our hypothesis, since geraniol and 10HDA were described in larval food of other bee species and could be one of the compounds of glandular secretions of *M. scutellaris*, we decided to also analyse the metabolites present in *M. scutellaris* larval food. We tentatively identified 369 metabolites and, the function enrichment analysis indicated significant enrichment for metabolites related to lactose degradation and galactose metabolism (P < 0.05). These results are expected, since larval food is the only energy source from the embryonic phase until the hatching of the imago, and are in accordance with the results found in food analysis of *Melipona quadrifasciata*^64^. Moreover, enzymes involved in carbohydrate metabolism, including α-galactosidase and ß-galactosidase, have been formerly identified in *M. scutellaris* larval food^66^.

Surprisingly, different larval food samples shared few metabolites, indicating a great diversity of tentatively identified compounds. The metabolites shared by more than two samples are related to carbohydrate metabolism. One of the two metabolites shared for all samples is 2,3-butanediol is produced by fermentation by a variety of microorganisms^67^. Stingless bees’ larval food is rich in bacteria, yeasts, and fungi, which ferment the food and produce a wide range of secondary metabolites^68–70^. The diversity of compounds may be the result of different levels of metabolism by different microbiotas present in each brood cell.

Again, we were not able to identify metabolites directly linked to geraniol, 10HDA and JH metabolisms in *M. scutellaris* larval food. This finding reinforces our hypothesis that neither geraniol nor 10HDA, alone, are capable of inducing queen phenotype in this species. In *Melipona compressipes*, it was demonstrated that 25% of larvae reared in mixed larval food (food collected from different combs, homogenised, and redistributed) differentiate in queens; proving that there is no role for differential feeding in caste differentiation in these bees^71^. Indeed, a complex mechanism such as female polyphenism caste differentiation should not rely on a single molecule present in larval food.

Finally, we evaluated the effects of 10HDA topical application in *M. scutellaris* larvae to confirm if this HDACi is not involved in caste polyphenism in these bees, and as an additional strategy to investigate the role of histone acetylation in this process. None of the 10HDA-treatments was effective in promoting the differentiation of the larvae in queens. Critical periods in development are represented by intervals in which an organism is especially sensitive to environmental stimuli. In insect caste differentiation, these periods indicate increased sensitivity to some regulatory hormones, such as JH^24^. Topical application of JH in *Melipona* larvae, in the temporal window that corresponds to the end of the third larval instar (stages from L3.3 to LPD), leads to the differentiation of the larvae in queens; the treatment before that period causes mortality and; after that window, does not promote differentiation^13^. In this study, we performed 10HDA-treatments in the same time interval in which *M. scutellaris* larvae are sensitive to JH application, however, they were not responsive to the HDACi treatment. Moreover, the highest 10HDA concentration tested induced high mortality (62.77%) on the treated larvae, and this compound was not found neither in *M. scutellaris* larvae nor in larval food, suggesting that there is no target for 10HDA in this species. Therefore, 10HDA is not a caste-differentiation factor in *M. scutellaris* stingless bees.

Epigenetic modifications possibly represent an association between genotype and environment in the development of divergent phenotypes in *Melipona* females^48^. Our expression data of genes related to acetylation machinery reinforce previous findings of our group^48,49^ that caste differentiation in *M. scutellaris* may be accomplished through an association of epigenetic mechanisms and endocrine signalling. Moreover, we showed that despite histone acetylation seems to be involved in *M. scutellaris* polyphenism, 10HDA alone is not able to promote queen differentiation in this stingless bee species.

## Material and methods

### Bees and larval food

Larvae, pupae, adult bees, and larval food were collected from *Melipona scutellaris* colonies kept in the Meliponary of Federal University of Uberlândia, Uberlândia, MG, Brazil. The classification of developmental stages followed previously described parameters^53,72^ (Supplementary Table S7). For analysis of metabolites and volatile compounds, samples were collected on the same day and from a single colony.

### RNA extraction, cDNA synthesis and RT-qPCR assays

Total RNA was extracted using TRIzol (Invitrogen) following the manufacturer’s recommendations. Each sample consisted of individual whole-body larvae, pupae or newly emerged bees. RNA was treated with 10U of DNAse I RNAse free (Promega), following the manufacturer's recommendations. RNA concentrations and quality were measured in spectrophotometer at 260 nm (ND-1000 Spectrophotometer). From the DNase-treated RNA, 1 μg were used as template RNA to synthetize the first strand cDNA using Oligo dT (15) (Invitrogen) and M-MLV Reverse Transcriptase enzyme (Promega) according to the manufacturer’s protocol. Each sample was analysed in duplicate with a quantitative PCR assay (QuantiNova SYBR Green PCR Kit, Qiagen) to evaluate the transcript levels of two HDAC genes (*hdac1* and *hdac4*) and one HAT gene (*kat2a*) using 2.5 pmol of each specific primer. The following cycling conditions were used in the RT-qPCR assays: 50 °C for 2 min, 95 °C for 10 min, 40 cycles of 95 °C for 15 s and 60 °C for 1 min. Simultaneously, the ribosomal protein *rpl32* gene was accordingly analysed for sample normalization; the gene has previously been validated for use as endogenous control for stingless bees RT-qPCR assays^73^. Product specificity was validated for all samples by running a melting curve analysis after the last amplification step. Relative expression values were calculated using the 2^−ΔΔCT^ equation^74^. Primer sequences for RT-qPCR reactions were as follows: *rpl32*-F: 5′ CGTCATATGTTGCCAACTGGT 3′, *rpl32*-R: 5′ TTGAGCACGTTCAACAATGG 3′; *hdac1*-F: 5′ GGTCTGTAGCTGCTGCTGTGA 3′, *hdac1*-R: 5′ GCATGATGTAAACCACCACCCT 3′; *hdac4*-F: 5′ AAGAATGCGTTCGGGCTTG 3′, *hdac4*-R: 5′ CGCGTTTTGGCAAGGTATCC 3′; *kat2a*-F: 5′ TTACGAAGGGGCAACACTGA 3′, *kat2a*-R: 5′ CTTCCGTATGACAGCCGTA 3’.

### Analysis of metabolites of larvae and larval food

The metabolomic analysis of larvae was performed with larvae of the second (L2) and third larval instar (L3.3 and LD) in sample and technical triplicates. For the larval food metabolomics, brood cells containing eggs were uncapped, the eggs removed and the food from each cell was individually transferred to a 1.5 mL microtube. The analysis of the food was performed in sample quintuplicate and technical triplicate. Sample preparation consisted in addition of 5 µL of internal standard D27 Myristic acid (3 mg/mL) to each sample, and then, they were dried in SpeedVac Vacuum (Thermo Fisher Scientific) for 18 h. For derivatization, dried metabolites were solubilized in 20 μL of methoxylamine (40 mg/mL in pyridine), spiked with 3μL of FAME (Fatty acid methyl ester) and incubated for 16 h at 25 °C and 650 rpm agitation. Then, 90 μL of MSTFA (N-Trimethylsilyl-N-methyl trifluoroacetamide) with 1% TMCS (Trimethylchlorosilane) was added. Samples were incubated for 1 h at 25 °C, followed by centrifugation at 15,800*g* for 10 min at room temperature. After derivatization, the supernatants were transferred to glass inserts and, 1 μL of each sample was injected randomly into an Agilent 7890B GC system operated in splitless mode, accordingly to the protocol established by Titan et al.^75^ with few modifications. A DB5-MS + 10 m Duraguard capillary column (Agilent #122-5532G) within which helium carrier gas flowed at a rate of 0.82 mL/min, was applied for metabolite separation. The injector temperature was set at 250 ºC. The column temperature was held at 60 °C for 1 min, and then increased to 310 °C at a rate of 10 °C/min for 37 min. The column effluent was introduced into the ion source of an Agilent 5977A mass selective detector. The detector operated in the electron impact ionization mode (70 eV) and mass spectra were recorded after a solvent delay of 6.5 min with 2.9 scans per second, starting at mass 50 and ending at mass 550, with step size of 0.1 m/z. The MS quadrupole temperature was set at 180 °C and the ion source temperature was set at 280 °C. Data were deconvoluted with RT size window of 75 and 100, SNR threshold of 1, extraction window m/z delta of 0.3 AMU on left and 0.7 on right. Tentatively identification of compounds was made comparing the mass spectra and retention time (RT) of all detected compounds with the Agilent Fiehn GC/MS Metabolomics RTL Library (Version A.02.02). The metabolites that were not identified in the Agilent Fiehn GC/MS Metabolomics RTL Library were searched in the National Institute of Standards and Technology (NIST) library 11 (2014) using Unknowns—Agilent MassHunter Workstation Quantitative Analysis (Version B.06.00).

### Treatment with 10-hydroxy-2E-decenoic acid

Pre-defecating (LPD) and L3.3 larvae of *Melipona scutellaris* were collected from combs and transferred to petri dishes where they were topically treated with a single dose of 1 µl of 10-hydroxy-2E-decenoic acid (10HDA, Cayaman Chemicals, CAS Number 14113-05-4) diluted in ethanol, in the following concentrations: 1,88 mM; 5 mM; 15 mM; 30 mM and 107 mM. Control groups were composed by larvae treated with 1 µl of ethanol (vehicle) and larvae that did not receive treatment. The larvae were kept at 29–30 °C with 75% relative humidity obtained with NaCl saturated solution until adult hatching^76^. The individuals were classified by caste when they reached the pupae phase of white eye and white body (Pw). The worker or queen phenotype was identified by means of morphological characteristics already defined in previous works^12^.

### Statistical analysis

Statistical analysis of gene expression were performed on GraphPad Prism (Version 8.00). As the data presented an asymmetric distribution, Kruskal–Wallis test was performed with a post-hoc Dunn’s multiple comparisons test. The hypothesis of equality was rejected for P < 0.05. For comparisons between castes, Two-Way-ANOVA was performed with a post-hoc Sidak's multiple comparisons test. The relative mRNA levels were presented as mean ± SEM (standard error of the mean).

Metabolomics analysis were performed on the Metaboanalyst software (Version 5.00). The concentrations of metabolites were first normalized by the mass (g) of each larva / sample. Then, the values were normalized by the mean and standard deviation of each group. Significant metabolites were identified by.

Significance Analysis of Microarrays (SAM). In Correlation Heatmap, Pearson R were applied for distance measure; in Hierarchical Clustering Dendrogram, Euclidean distance was applied, and Ward was used as a clustering algorithm.

## Supplementary Information

Supplementary Information 1.

Supplementary Information 2.

Supplementary Information 3.

Supplementary Information 4.

Supplementary Information 5.

Supplementary Information 6.

Supplementary Information 7.

Supplementary Information 8.

## Data Availability

The datasets generated during and/or analysed during the current study are available from the corresponding author on reasonable request.
