# Supplementary material for: 10-hydroxy-2E-decenoic acid (10HDA) does not promote caste differentiation in Melipona scutellaris stingless bees
Source: Sci Rep. 2021 May 10;11:9882. doi: 10.1038/s41598-021-89212-5 (PMC8110752; doi:10.1038/s41598-021-89212-5)
Supplement: Supplementary file 7 — Supplementary Information 7. [file 41598_2021_89212_MOESM7_ESM.pdf]

10-hydroxy-2E-decenoic acid (10HDA) does not promote caste differentiation in *Melipona scutellaris* stingless bees

Luiza Diniz Ferreira Borges[1*], Letícia Leandro Batista[1], Serena Mares Malta[1], Tamiris Sabrina Rodrigues[1], Jéssica Regina da Costa Silva[1], Gabriela Venturini[2], Alexandre da Costa Pereira[2], Pedro Henrique Gonçalves Guedes[1], Carlos Ueira-Vieira[1], Ana Maria Bonetti[1*]

Supplementary Table S7: *Melipona scutellaris* developmental stages. The classification of developmental stages followed previously described parameters [53,72].

Stage	Classification
L1	1st instar larva
L2	2nd instar larva
L3.1	3rd instar larva: large quantity of liquid food; larvae with curved body and of pearly color
L3.2	3rd instar larval: slightly less food of more viscous consistency; the larvae continue curved and of pearly color
L3.3	3rd instar larva: little food of solid consistency left in the brood cells; the larvae are of bright pearly color
LPD	3rd instar pre-defecating larva: brood cells without food; the defecation process has not yet started; the larvae are slightly bent with head pointing upward and still are of bright pearly color
LD	3rd instar defecating larva: brood combs without food; the larvae started to void the gut; they are straight with the head pointing toward upward; color is now whitish
Pw	White-eyed pupa
Pp	Pink-eyed pupa
Pb	Brown-eyed pupa with unpigmented body
Pbl	Brown-eyed pupa with lighlty-pigmented body
Pbd	Brown-eyed pupa with dark-pigmented body
NE	Newly-emerged adult
