# Supplementary material for: 10-hydroxy-2E-decenoic acid (10HDA) does not promote caste differentiation in Melipona scutellaris stingless bees
Source: Sci Rep. 2021 May 10;11:9882. doi: 10.1038/s41598-021-89212-5 (PMC8110752; doi:10.1038/s41598-021-89212-5)

10-hydroxy-2E-decenoic acid (10HDA) does not promote caste differentiation in *Melipona scutellaris* stingless bees

Luiza Diniz Ferreira Borges[1*], Letícia Leandro Batista[1], Serena Mares Malta[1], Tamiris Sabrina Rodrigues[1], Jéssica Regina da Costa Silva[1], Gabriela Venturini[2], Alexandre da Costa Pereira[2], Pedro Henrique Gonçalves Guedes[1], Carlos Ueira-Vieira[1], Ana Maria Bonetti[1*]

Supplementary Figure S1: Significance Analysis of Microarrays (SAM) of metabolites of *Melipona scutellaris* larvae. SAM shows 45 significant metabolites tentatively identified in the larvae. These are presented in Supplementary Table S2. FDR = false discovery rate.

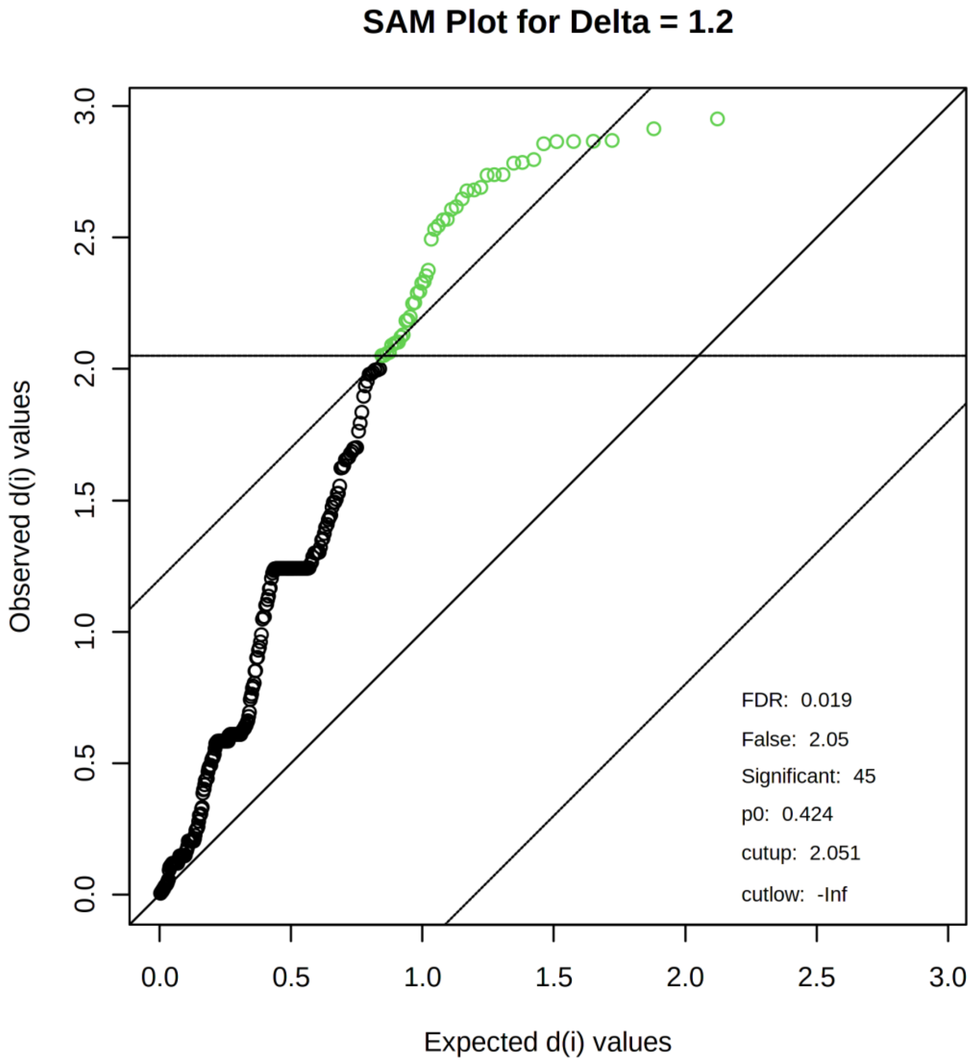

Supplementary Figure S2: Metabolites of *Melipona scutellaris* larval food.

Venn diagram of shared metabolites between larval food samples.

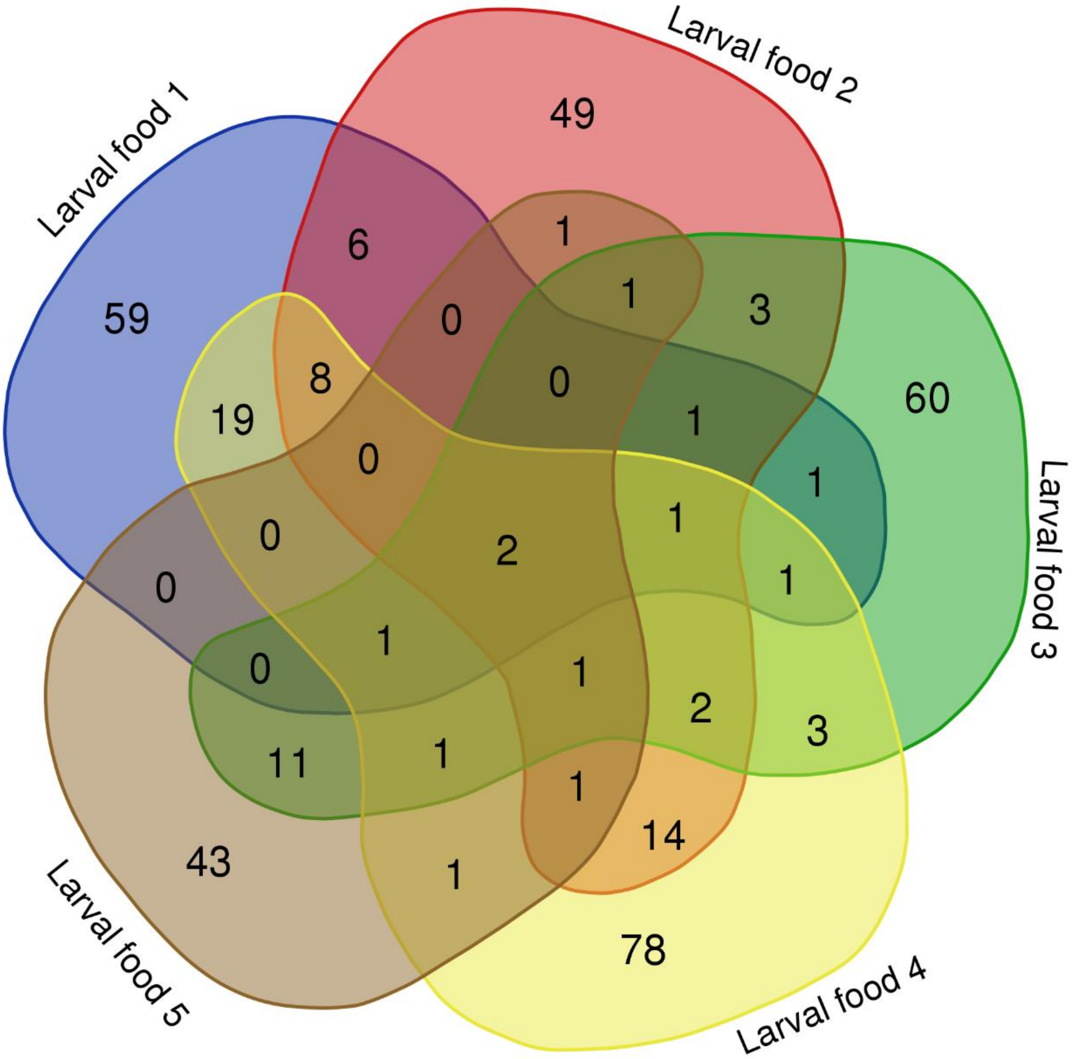

Supplement: Supplementary file 8 — Supplementary Information 8. [file 41598_2021_89212_MOESM8_ESM.pdf]
